# Proteomic analysis reveals dysregulation of peripheral blood neutrophils in patients with Multiple Sclerosis

**DOI:** 10.1093/cei/uxae115

**Published:** 2025-01-16

**Authors:** Katie J Smith, Zachary Lim, Sonja Vermeren, Veronique E Miron, Sarah Dimeloe, Donald J Davidson, Anna Williams, Emily Gwyer Findlay

**Affiliations:** Centre for Inflammation Research, Institute for Regeneration and Repair, University of Edinburgh, Edinburgh, UK; School of Biological Sciences, University of Southampton, Southampton, UK; Centre for Inflammation Research, Institute for Regeneration and Repair, University of Edinburgh, Edinburgh, UK; United Kingdom Dementia Research Institute at The University of Edinburgh, Edinburgh, UK; Keenan Research Centre for Biomedical Sciences at St. Michael’s Hospital, Unity Health Toronto, Toronto, ON, Canada; Department of Immunology, University of Toronto, Toronto, ON, Canada; Institute of Immunology and Immunotherapy, University of Birmingham, Birmingham, UK; Centre for Inflammation Research, Institute for Regeneration and Repair, University of Edinburgh, Edinburgh, UK; Centre for Regenerative Medicine, Institute of Regeneration and Repair, University of Edinburgh, UK; Centre for Inflammation Research, Institute for Regeneration and Repair, University of Edinburgh, Edinburgh, UK; School of Biological Sciences, University of Southampton, Southampton, UK

**Keywords:** neutrophils, proteomics, T cells, Multiple Sclerosis, CD161

## Abstract

Multiple Sclerosis (MS) is a complex auto-inflammatory disease affecting the brain and spinal cord, which results in axonal de-myelination and symptoms including fatigue, pain, and difficulties with vision and mobility. The involvement of the immune system in the pathology of MS is well established, particularly the adaptive T cell response, and there has been a particular focus on the IL-17-producing subset of Th17 cells and their role in driving disease. However, the importance of innate immune cells has not been so well characterized. Here we focussed on neutrophils, which are innate immune cells and rapid responders to inflammation, and which have recently been linked to other chronic autoimmune conditions. Multiple strands of evidence in patients with MS and in mice with the experimental autoimmune encephalomyelitis MS model suggest neutrophils may play a role in driving MS inflammation. Here, we performed proteomic analysis on neutrophils from patients with MS and healthy donors, revealing striking differences. In particular, granule proteins were significantly more abundant in the MS neutrophils compared to the healthy controls, with a particular overabundance of proteins in primary and secondary granules. In addition, members of the MAVS signalling pathway were differently regulated compared to healthy donor cells. Finally, we find that MS neutrophils do not suppress T cell activation equivalently to healthy neutrophils, and in particular are unable to suppress expression of CD161 on the T cells, indicative of a suppression of Th17 differentiation. We propose that neutrophil dysregulation in MS may contribute to dysfunctional T cell responses.

## Teaser

Analysis of peripheral blood neutrophils from patients with Multiple Sclerosis shows many differences compared to healthy cells. In particular, MS neutrophils have dysregulated granule protein expression and are less able to suppress T-cell activation.

## Graphical Abstract

**Graphical Abstract:**
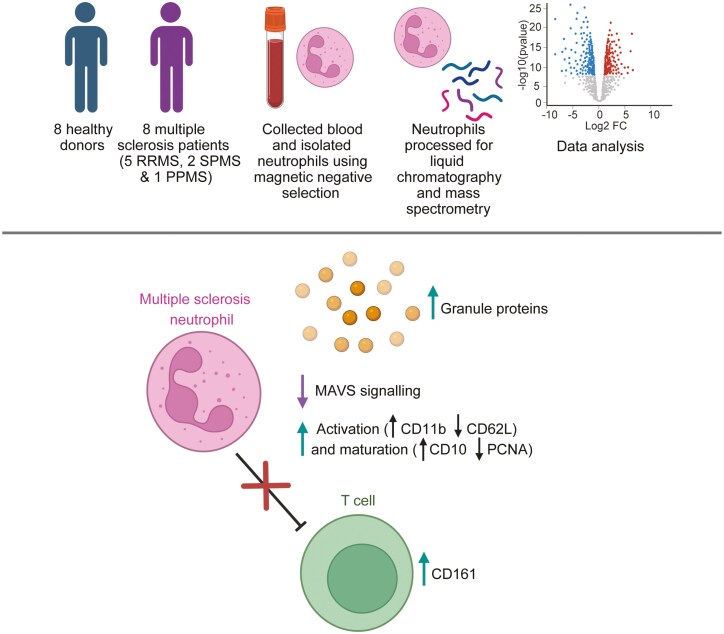

## Introduction

Multiple Sclerosis (MS) is an auto-inflammatory disease affecting the central nervous system. The development and pathology of MS are complex, with early inflammation leading to later neuro-degeneration, axonal demyelination and axonal loss. The contributions of adaptive immunity such as T cells (in particular Th1 and Th17 cells and their cytokines) to the inflammatory stage of MS development are well-established [1–7], but other contributors to inflammation in early MS are less understood. In particular, the role of innate immune cells such as neutrophils is unclear.

Neutrophils are the most abundant leukocyte in human blood and the first incoming responders to infectious and sterile inflammation. Owing to their rapid recruitment during the initiation of inflammation, short lifespan, and rapid clearance following death, the role of neutrophils in driving chronic diseases such as MS was long underestimated. More recently, however, neutrophil half-life estimations have been considerably extended [8]; continual neutrophilia has been found to be a feature of other chronic autoimmune and autoinflammatory conditions [9, 10]; and their interactions with adaptive immune cells such as T cells has been revealed to be specific and complex [11–16]. An interest in the role of neutrophils in long-term autoimmune diseases like MS is therefore developing.

It is now well established that an increased systemic neutrophil-to-lymphocyte ratio is a marker of MS disease activity [17–20]. Locally, neutrophils have been found in active brain lesions [12, 21]. Not only the frequency of neutrophils but also their phenotype is changed with disease—peripheral blood neutrophils from patients with MS are typically more activated, produce more reactive oxygen species (ROS) and produce neutrophil extracellular traps more rapidly than those from healthy donors [17, 22].

Correlative studies suggest reducing neutrophil numbers would be beneficial in disease. A positive response to treatment correlates with neutropenia [23], and in contrast, GCSF therapy (which stimulates the production and emigration of immature neutrophils from the bone marrow) worsens disease exacerbations [24].

The mouse model of the inflammatory stage of MS, experimental autoimmune encephalomyelitis, also shows strong pathogenic roles for these cells. Mice depleted of neutrophils do not develop disease [25, 26]. Neutrophils isolated from the spinal cord during disease promote the activation and maturation of antigen-presenting cells [25]. We have also previously shown that neutrophils in the spinal cord and brain of affected mice direct a pathogenic T-cell response through the release of the granular antimicrobial peptide cathelicidin [12].

All of this work demonstrates a clear need for further investigation into neutrophils in patients with MS—yet no unbiased screening of these cells has yet been performed. Here, we undertook such an approach through proteomic analysis of rapidly isolated neutrophils from patients with MS and healthy donors. Our results show a profound and consistent dysregulation of peripheral blood neutrophils in patients diagnosed with MS, with neutrophils appearing more mature, having consistently more abundant antimicrobial peptides, and altered expression of members of the mitochondrial antiviral-signalling protein (MAVS) signalling pathway. Co-culture of these neutrophils with T cells showed that T cells contacting healthy neutrophils are suppressed in their activation and less likely to express markers of Th17 differentiation, while T cells contacting MS neutrophils do not show this suppression. This suggests a route by which dysregulated neutrophils may lead to differences in adaptive immune responses in autoimmune diseases.

## Methods

### Patients and ethical agreements

The study was approved by Lothian Bioresource under agreement SR1323. Patients with MS attending the Anne Rowling Regenerative Neurology Clinic (Edinburgh, Scotland) for post-diagnosis clinical meetings were enrolled in the study, with full written consent, by clinical staff. Exclusion criteria were: under the age of 18; over the age of 65; and on any disease-modifying drug therapies. Healthy donor blood was collected under ethical agreement 21-EMREC-041, with full written consent (Table 1).

**Table 1:** Proteomics donor information table

Donor	Age	Sex	Diagnosis
MS1	31–40	F	RRMS
MS2	61–70	F	RRMS
MS3	31–40	F	SPMS
MS4	31–40	F	RRMS
MS5	41–50	F	RRMS
MS6	51–60	M	RRMS
MS7	51–60	F	PPMS
MS8	51–60	F	SPMS
HD1	41–50	F	—
HD2	21–30	F	—
HD3	41–50	F	—
HD4	41–50	M	—
HD5	21–30	F	—
HD6	41–50	F	—
HD7	31–40	F	—
HD8	21–30	F	—

MS, multiple sclerosis; HD, healthy donor; RRMS, relapsing-remitting multiple sclerosis; SPMS, secondary progressive multiple sclerosis; PPMS, primary progressive multiple sclerosis

### Isolation of neutrophils and T cells

Highly purified neutrophils and CD3^+^ T cells were immediately isolated from fresh blood using immunomagnetic negative separation. The Stem Cell Technologies direct neutrophil isolation kit (StemCell Technologies, #17957) and Stem Cell Human T-cell isolation kit (StemCell Technologies, #17951) were used respectively, as per the manufacturer’s instructions. The purity of both cell populations was routinely >95%.

### Flow cytometry

Cells were stained with either 1 μg/ml DAPI (Invitrogen #D1306) or 1:1000 live/dead yellow (Invitrogen #L-34959) diluted in 1XPBS, or 1:150 Zombie NIR Fixable Viability Kit (Biolegend, #423105) diluted in 1XPBS for 20 minutes at room temperature, protected from light. Surface markers (listed below) were stained for 30 minutes at 4°C in 1XPBS, protected from light. Samples were run on a BD Biosciences LSR Fortessa cytometer and data was analysed using FlowJo software, version 10 (BD Biosciences).

Antibodies used include: CD3 FITC (clone HIT3a, Biolegend, #300305, 1:200), CD4 PeCy7 (clone A161A1, Biolegend, #357409, 1:200), CD8 APC (clone SK1, Biolegend, #344721, 1:200), CD62L PeDazzle (clone DREG-56, Biolegend, #304805, 1:200), CD62L AF488 (clone DREG-56, Biolegend, #304816, 1:150), CD161 APC (clone HP-3G10, Biolegend, #339911, 1:200), CD11b APC/fire (clone CBRM1/5, Biolegend, #301419, 1:150), CD44 BV510 (clone IM7, Biolegend, #103049, 1:400).

### Preparation of neutrophils for proteomic analysis

Sample preparations and mass spectrometry were performed as a service by the Fingerprints Proteomics facility, at the University of Dundee.

Following isolation, neutrophils were centrifuged at 10,000g for 8 minutes, the supernatant was removed and discarded, and the neutrophil pellet was snap-frozen and stored at −80°C until mass spectrometry was carried out. Pellets were then lysed in a buffer containing 5% SDS and 50 mM triethylammonium bicarbonate and sonicated for 30 seconds on/30 seconds off at 50% amplitude. 20 mM dithiothreitol was added and cells were boiled at 95°C for 10 minutes. Once cooled, 40 mM iodoacetamide was added for 30 minutes at room temperature. Proteins were isolated with S-TRAP columns. Cells were digested with trypsin overnight at 37°C before more trypsin was added for 6 hours and proteins collected by elution from the columns with 50 mM triethylammonium bicarbonate, 0.2% aqueous formic acid and 50% aqueous acetonitrile with 0.2% formic acid. Protein concentration was determined using the EZQ protein quantitation kit (Invitrogen, #R33200).

### Liquid chromatography and mass spectrometry (LC/MS)

Neutrophil proteins were quantified and normalized using a microBSA assay. A total of 1.5 µg of peptide was analyzed per sample. Samples were injected onto a nanoscale C18 reverse-phase chromatography system (UltiMate 3000 RSLC nano, Thermo Scientific) and then electrosprayed into a Q Exactive HF-X Mass Spectrometer (Thermo Scientific). For liquid chromatography, buffers were as follows: buffer A (0.1% formic acid in Milli-Q water [v/v]) and buffer B (80% acetonitrile and 0.1% formic acid in Milli-Q water [v/v]). Sample were loaded at 10 μL/min onto a trap column (100 μm × 2 cm, PepMap nanoViper C18 column, 5 μm, 100 Å, Thermo Scientific) equilibrated in 0.1% trifluoroacetic acid (TFA). The trap column was washed for 3 minutes at the same flow rate with 0.1% TFA then switched in-line with a Thermo Scientific, resolving the C18 column (75 μm × 50 cm, PepMap RSLC C18 column, 2 μm, 100 Å). The peptides were eluted from the column at a constant flow rate of 300 nl/min with a linear gradient from 3% buffer B to 6% buffer B in 5 minutes, then from 6% buffer B to 35% buffer B in 115 minutes, and finally to 80% buffer B within 7 minutes. The column was then washed with 80% buffer B for 4 minutes and re-equilibrated in 35% buffer B for 5 minutes. Two blanks were run between each sample to reduce carry-over. The column was kept at a constant temperature of 40°C.

The data was acquired using an easy spray source operated in positive mode with spray voltage at 1.950 kV, and the ion transfer tube temperature at 250°C. The MS was operated in DIA mode. A scan cycle comprised a full MS scan (m/z range from 350 to 1650), with RF lens at 40%, AGC target set to custom, normalized AGC target at 300, maximum injection time mode set to custom, maximum injection time at 20 ms and source fragmentation disabled. MS survey scan was followed by MS/MS DIA scan events using the following parameters: multiplex ions set to false, collision energy mode set to stepped, collision energy type set to normalized, HCD collision energies set to 25.5, 27, and 30, orbitrap resolution 30 000, first mass 200, RF lens 40, AGC target set to custom, normalized AGC target 3000, maximum injection time 55 ms.

Data analysis was carried out using Spectonaut (version 16.2.220903.53000, Biognosys, AG). The directDIA workflow, using the default settings (BGS Factory Settings) with the following modifications were used: decoy generation set to mutated; Protein LFQ Method was set to QUANT 2.0 (SN Standard) and Precursor Filtering set to Identified (Qvalue); Precursor Qvalue Cutoff and Protein Qvalue Cutoff (Experimental) set to 0.01; Precursor PEP Cutoff set to 0.1 and Protein Qvalue Cutoff (Run) set to 0.05. The data were normalized on the global median. For the Pulsar search the settings were: maximum of two missed trypsin cleavages; PSM, Protein and Peptide FDR levels set to 0.01; scanning range set to 300–1800 m/z and Relative Intensity (Minimum) set to 5%; cysteine carbamidomethylation set as fixed modification and acetyl (Protein N-term), deoxidation (methionine, tryptophan), deamidation (asparagine, glutamine) and oxidation of methionine set as variable modifications. The database used was H.sapiens proteome downloaded from niport.org on 2021-05-11 (77027 entries).

### *In vitro* co-culture system

A total of 75 000 T cells and 225 000 neutrophils were cultured together in one well of a 96-well plate. MS patient and healthy donor T cells were plated alone, or co-cultured with neutrophils, with 0.5µl/ml CD3/CD28/CD2 T-cell activator (STEMCELL Technologies, #10970). Cells were incubated at 37°C for 24 hours.

### Software used

The volcano plot (Fig. 2A) was generated in R using the ggplot2 (v3.5.0) and ggrepel (v0.9.5) package with default parameters. Upregulated genes were defined as mlog10p value of >1.3 and a log 2-fold change value of >1 and downregulated genes were defined as mlog10p value of >1.3 and a log 2-fold change value of <−1. Pathway analysis was performed in R using the clusterProfiler (v4.10.1) package with the enrichGO function with a *P*-value cut off as 0.05.

The graphical abstract was created using Biorender, and graphing and statistical analysis was performed using GraphPad Prism.

## Results

No unbiased analysis of neutrophils in MS has yet been performed, although there is mounting evidence that the cells are dysregulated in disease. To perform these experiments, we collected peripheral blood from patients with MS and from control donors.

### Peripheral blood neutrophils are activated in patients with MS

**Figure 1:**
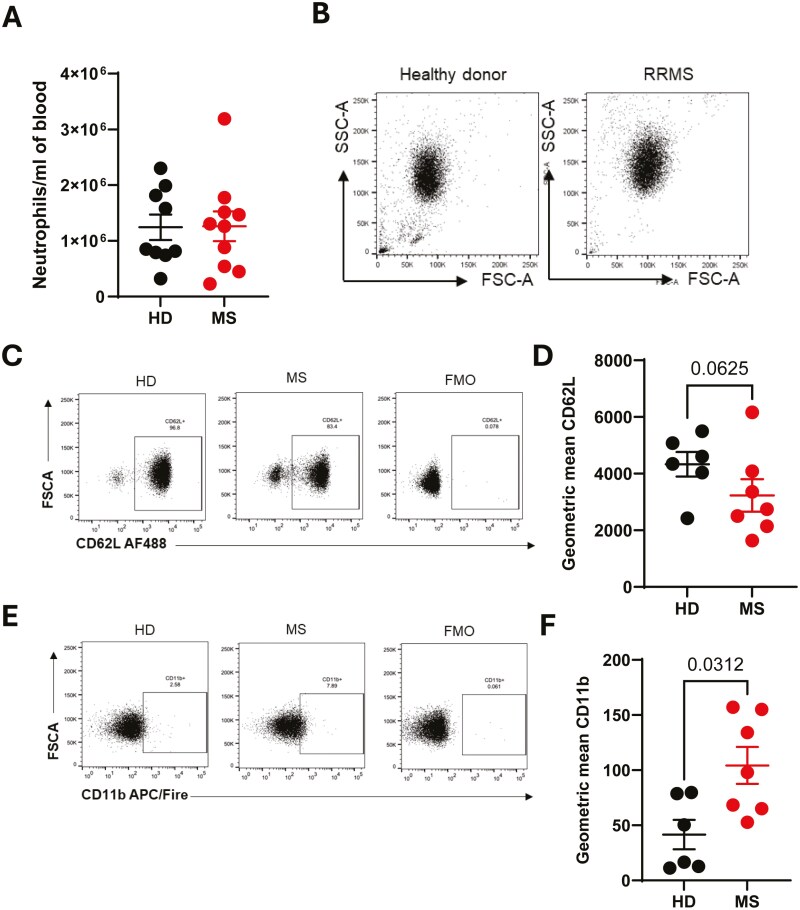
**Peripheral blood neutrophils from patients with multiple sclerosis are more activated than cells from healthy donors.** Peripheral blood was drawn from patients with multiple sclerosis (MS, red) and healthy donors (HD, black). Neutrophils were isolated immediately via negative magnetic selection and (A) cell counts performed using a haemocytometer. (B-F) Neutrophils were assessed by flow cytometry. Representative forward scatter and side scatter shown (B). Activation was measured by CD62L loss (C, D) and CD11b gain (E, F). HD – healthy donor; MS – multiple sclerosis; FMO – fluorescent minus one. N values: 6-10. Statistical test: D, F – Wilcoxon matched-pairs test.

There were no differences in total neutrophil number or size / granularity in peripheral blood between the groups (Fig. 1A, B). First, we analysed the cell surface phenotype of the neutrophils using flow cytometry for indicators of increased activation. Neutrophils from patients with MS showed lower expression of surface CD62L (Fig. 1C, D) and higher CD11b expression (Fig. 1E, F) than healthy donor cells, both indicative of increased activation. These data suggested that circulating neutrophils are activated in patients with MS, supporting a previous report [17], which also demonstrated the activation of MS neutrophils.

These initial results confirmed the importance of performing an unbiased, larger-scale analysis of these cells. Since neutrophils have comparatively low transcriptional activity, with many of their most abundant proteins stored in granules, we opted to use proteomics rather than RNA sequencing for their analysis; indeed, a number of recent studies have demonstrated the value of this technique in other diseases [27–29]. We therefore progressed by performing mass spectrometry analysis of proteins on frozen neutrophil pellets from our 16 samples.

### Neutrophils from patients with MS are activated and mature

**Figure 2:**
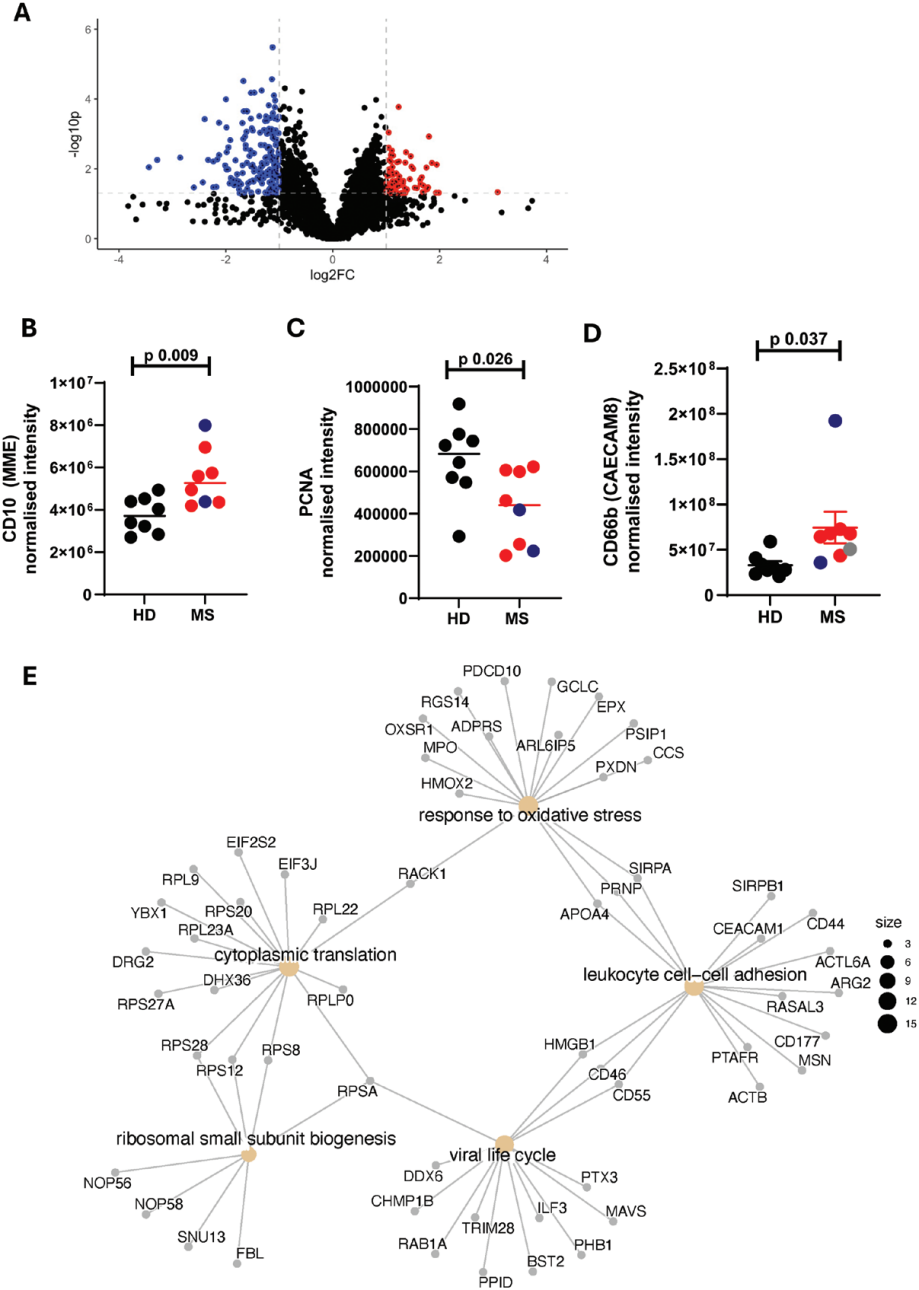
**Neutrophils from patients with MS are more mature.** Peripheral blood was drawn from patients (Multiple Sclerosis, MS) and healthy donors (HD). Neutrophils were isolated immediately via negative magnetic selection, centrifuged and cell pellets were snap-frozen. Cell pellets were analysed via LC/MS for protein abundance. (A) Unbiased volcano analysis of upregulated (red) and downregulated (blue) proteins in MS neutrophils compared to healthy donor cells defined by –log10pvalue of <1.3 and a log2fold change of > 1 or < -1, represented by the dotted lines. (B, C, D) Neutrophil maturity was assessed via CD10, PCNA and CD66b abundance in RRMS (red), SPMS (blue), PPMS (grey) and HD (black). (E) Pathway analysis of differentially expressed proteins in MS neutrophils compared to healthy donor neutrophils. Size of dots represents number of differentially affected proteins in each pathway. FC - fold change. N values: 6-8. Statistical test: B, C - unpaired t test with Welch’s correction

A total of 3890 unique proteins were identified in the samples, with differences clearly observable between the two groups (Fig. 2A). As our initial studies had demonstrated that MS neutrophils were characterized by an activated phenotype, we first analyzed the differential expression of markers of neutrophil inflammation and maturation. Of particular interest, the surface protein CD10 (membrane metalloendopeptidase) was upregulated in MS neutrophils (146% of HD value on average, Fig. 2B). In addition, the proliferation marker PCNA (proliferating cell nuclear antigen) was downregulated in these samples (64% of average HD value, Fig. 2C) and CD66b expression, which can denote activation, was also increased (225% of average HD value, Fig. 2D). This expression pattern suggests increased priming and maturation of neutrophils in the periphery of patients with MS.

At the point of sample collection, we did not know which course of MS our donors had, but later analysis allowed us to examine this. We had five patients with the most common form, relapsing-remitting MS (RRMS, red symbols), in which exacerbations are followed by remission. Two patients had secondary progressive MS (SPMS, blue symbols), in which RRMS develops into progressive worsening of symptoms, and one had primary progressive MS (PPMS, grey symbol), in which the patient does not have any periods of relapse even at the start of the disease course. Our study sample was not powered to uncover differences between these disease courses and we have not analysed them separately, but they are denoted by different colours in all graphs.

Pathway analysis of differentially expressed proteins showed significant alterations in antiviral responses (*P* adj-0.033) and response to oxidative stress (*P* adj-0.033), as well as demonstrating the altered intensity of proteins related to translation (*P* adj-0.000000267), ribosomal small subunit biogenesis (*P* adj-0.033) and cell–cell adhesion (*P* adj-0.038; Fig. 2E).

### Antimicrobial peptides and proteins are more abundant in MS neutrophils

Patients with MS are at increased risk of infection, even if they are not on any disease-modifying therapy [30–32]. We hypothesized that one reason for this may be that their neutrophils have impaired responses to pathogens. We therefore analysed the dataset for proteins relating to innate immune and anti-infection processes.

**Figure 3:**
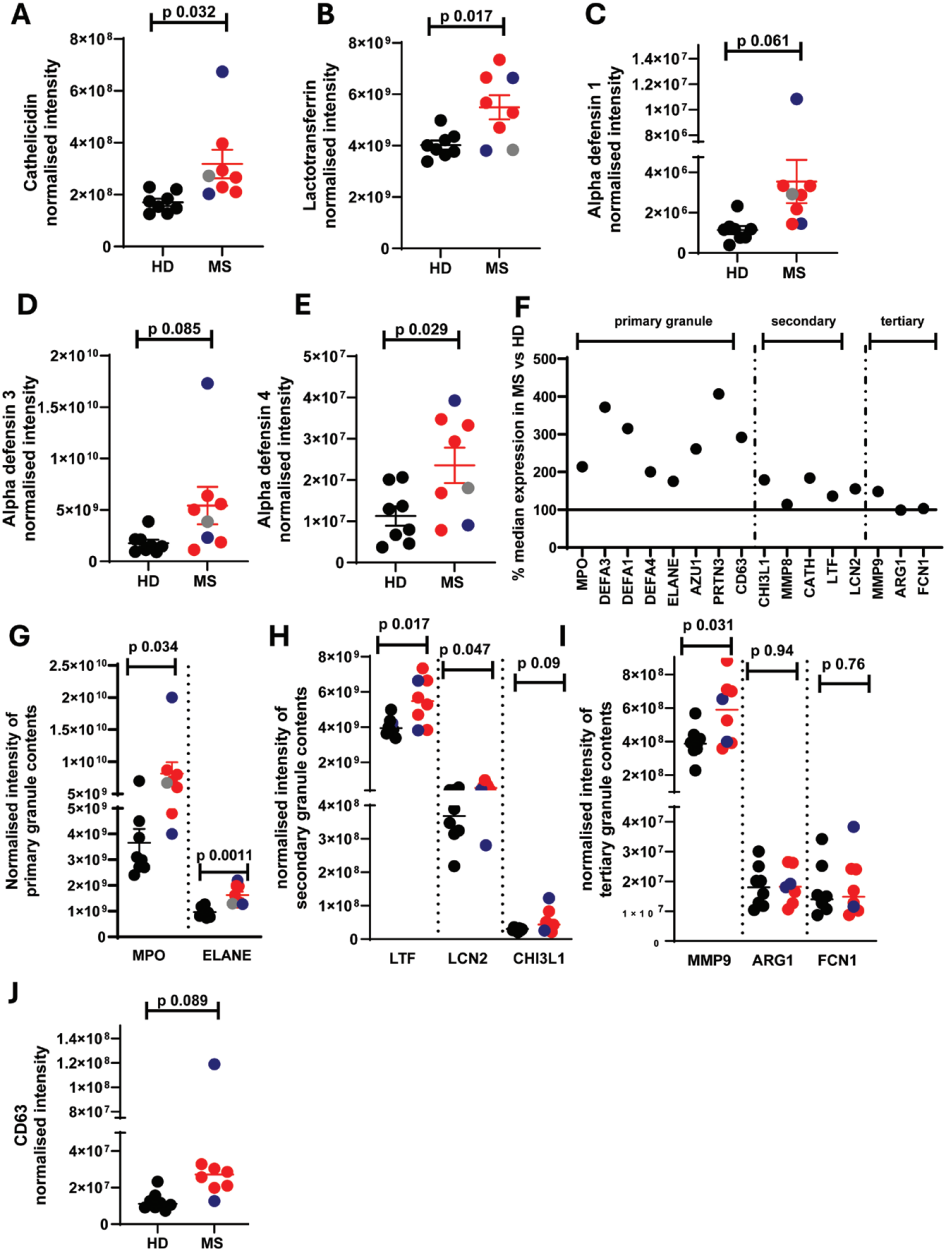
**Increased abundance of primary and secondary granule proteins in MS neutrophils.** Peripheral blood was drawn from patients (Multiple Sclerosis, MS; RRMS, red; SPMS, blue; or PPMS, grey) and healthy donors (HD, black). Neutrophils were isolated immediately via negative magnetic selection, centrifuged and cell pellets were snap-frozen. (A-E, G-J) Cell pellets were analysed via LC/MS for protein abundance. (F) granule protein abundance in MS neutrophils as a percentage of median healthy donor abundance, line at 100% expression. N values: 7-8. Statistical test- unpaired t test with Welch’s correction.

We noted first that antimicrobial peptides and proteins in MS neutrophils were significantly more abundant compared to HD cells. Cathelicidin (CATH), lactotransferrin (LTF), and multiple alpha-defensins (DEFA1, DEFA3, DEFA4) were all present at higher levels in MS neutrophils than healthy controls (Fig. 3A–E). Some results here appeared to be skewed by one SPMS patient with extremely high levels of granule proteins (shown in blue, Fig. 3A–D). We confirmed that RRMS patients (*n* = 5) had higher levels of these proteins by re-running statistical tests using only these patient values vs healthy controls. RRMS neutrophils did indeed have higher levels of cathelicidin (normalized intensity mean 2.8 × 10^8^ in MS vs 1.7 × 10^8^ in HD, *P* = 0.0048), alpha defensin 1 (mean 2.6 × 10^6^ vs 1.1 × 10^6^, *P* = 0.0024) and alpha defensin 3 (mean 3.99 × 10^9^ vs 1.76 × 10^9^, *P* 0.034) compared to healthy neutrophils.

Many antimicrobial peptides and proteins are stored in the cytoplasmic granules, of which there are three major types—primary, secondary and tertiary. We therefore extended our analysis to look at the granule protein composition in neutrophils from each group. Interestingly, there were large changes (% alteration shown in Fig. 3F, individual values in Fig. 3G–I) which differed for constituent proteins/peptides across the various types of granules. Primary granule contents were significantly more abundant in MS neutrophils compared to healthy neutrophils (Fig. 3F, G), with an average of all primary granule contents at 280% of the abundance in healthy donor cells, as demonstrated with myeloperoxidase (MPO) and elastase (ELANE; Fig. 3G). The secondary granule protein abundance was also increased compared to healthy cells (Fig. 3F, H), although less so than the primary granules, with a median abundance of 154% of the healthy value, demonstrated here with lactotransferrin (LTF), lipocalin 2 (LCN2) and chitinase 3-like protein 1 (CHI3L1; Fig. 3H). Interestingly, the tertiary granule contents were not more abundant in the MS neutrophils than the healthy cells (Fig. 3F, I), with the exception of MMP9—shown here also are arginase 1 (ARG1) and ficolin 1 (FCN1).

An increase in the abundance of granule proteins is intriguing as MS neutrophils are more activated, therefore we would have hypothesized that they undergo increased degranulation and consequently contain lower concentrations of granule proteins. In support of this, others have shown [17, 22] that CD63 expression, which can be used as a marker for the release of primary granules [33], is increased in MS neutrophils compared to healthy controls. We therefore examined the expression of CD63 in our dataset. We found it to be elevated in the MS neutrophils, although very variable between patients, with a mean of 292% of the expression seen in healthy neutrophils, (Fig. 3J), agreeing with this previous work. This data therefore suggests a combination of altered granule composition and degranulation rates in the MS neutrophils.

### Anti-infection responses are increased in MS neutrophils

**Figure 4:**
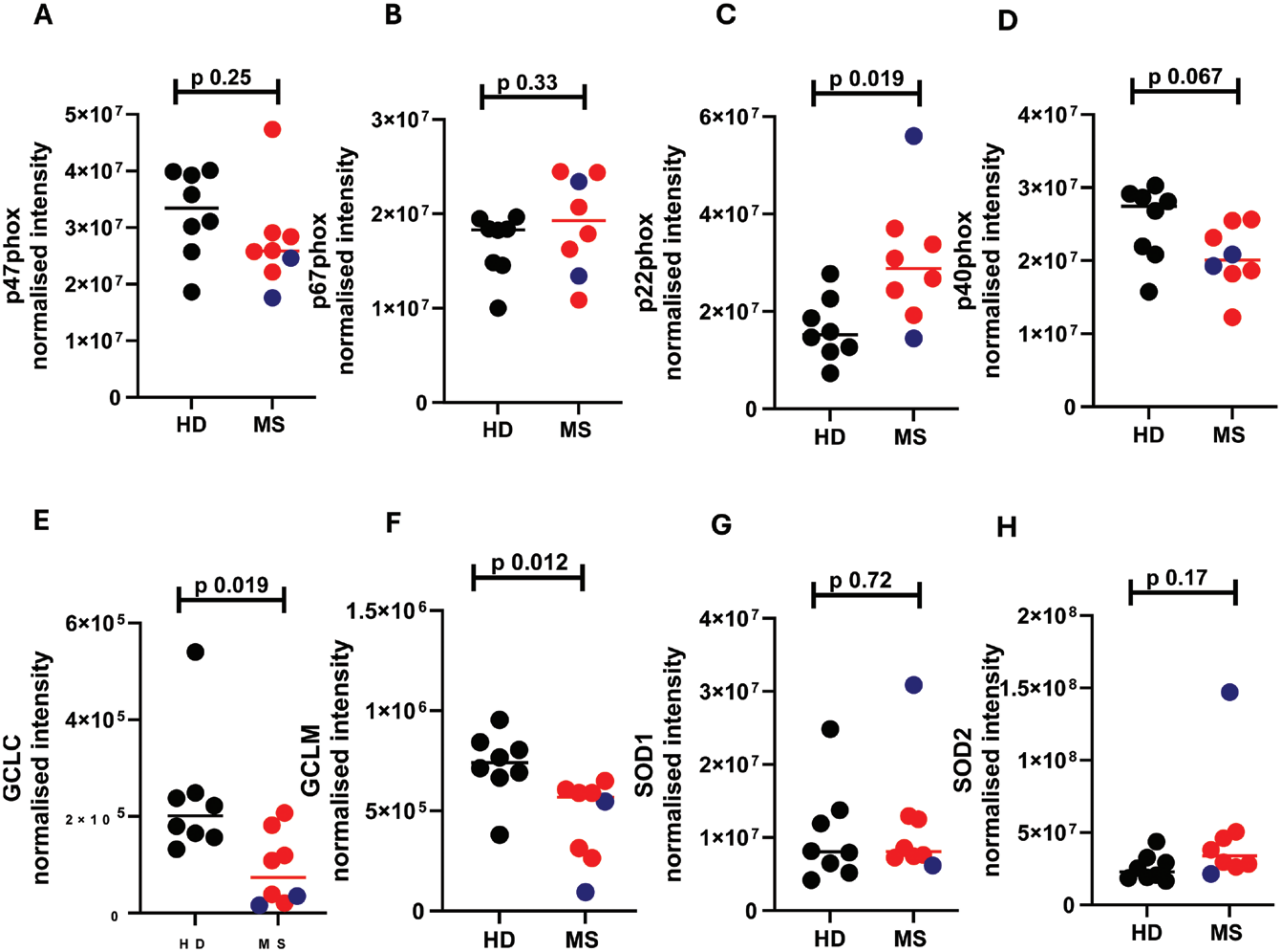
**Alterations in reactive oxygen pathways in MS neutrophils.** Peripheral blood was drawn from patients (Multiple Sclerosis, MS; RRMS, red; SPMS, blue; or PPMS, grey) and healthy donors (HD, black). Neutrophils were isolated immediately via negative magnetic selection, centrifuged and cell pellets were snap-frozen. (A-H) Cell pellets were analysed via LC/MS for protein abundance. N values: 7-8. Statistical test: A-H - unpaired t test with Welch’s correction

As our data are suggestive of dysregulated degranulation in MS neutrophils, we next wondered if other antimicrobial responses were altered in MS neutrophils, such as ROS. Firstly, the ‘response to oxidative stress’ was found to be altered in the pathway analysis (Fig. 2E). Secondly, we had noted previously that PCNA is expressed at lower levels in MS neutrophils (Fig. 2C). PCNA promotes the production of ROS [34] and so we hypothesized that MS neutrophils would produce lower concentrations of ROS. To test this hypothesis, we examined the expression of proteins involved in the generation of ROS: p47phox (NCF1), p67phox (NCF2), Gp91phox (NOX2), p22phox (CYBA), and p40phox (NCF4; Fig. 4). gp91phox was not found in the dataset. Of the others, p47phox and p67phox showed no difference between patients and healthy donors, while p22phox was significantly increased and p40phox was decreased in MS neutrophils (Fig. 4A–D).

To further investigate whether reactive oxygen pathways were altered in neutrophils from patients, we analyzed the abundance of glutamate-cysteine ligase (GCLC), glutamate-cysteine ligase regulatory subunit (GCLM), glutathione peroxidase 3 (GPX3), superoxide dismutase 1 (SOD1) and superoxide dismutase 2 (SOD2; Fig. 4E–H). Interestingly, GCLC ligase (Fig. 4E) and GCLM (Fig. 4F) were significantly downregulated in the MS neutrophils. No other proteins were altered between the two groups.

**Figure 5:**
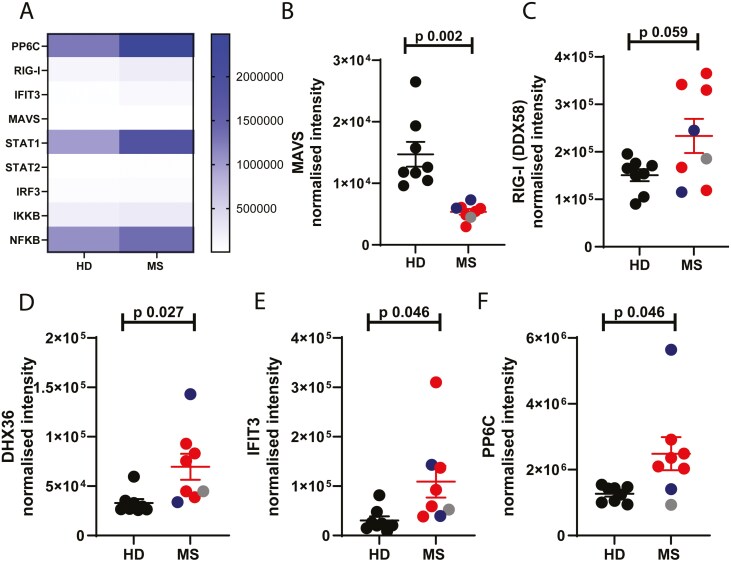
**Altered abundance of MAVS pathway members in MS neutrophils.** Peripheral blood was drawn from patients ((Multiple Sclerosis, MS; RRMS, red; SPMS, blue; or PPMS, grey) and healthy donors (HD, black). Neutrophils were isolated immediately via negative magnetic selection, centrifuged and cell pellets were snap-frozen. (A-F) Cell pellets were analysed via LC/MS for protein abundance. N values: 7-8. Statistical test: unpaired t test with Welch’s correction.

Next, we saw in the pathway analysis that MAVS came up as a key protein altered in abundance (Fig. 2E). We noted in the proteomic dataset that members of this MAVS signalling pathway were particularly affected in the patient cells (Fig. 5A). This is a key pathway through which interferons are produced, although it is not well characterized in neutrophils. In the MS neutrophils there was a large and significant decrease in the MAVS protein (Fig. 5B) and increases in the abundance of RIG-I (DDX58), DHX36, IFIT3 and PP6C (Fig. 5C–F).

**Figure 6:**
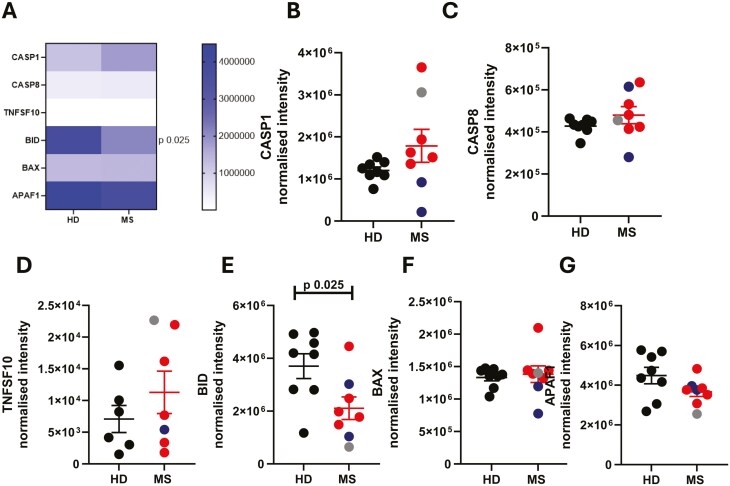
**Analysis of apoptosis-related proteins in MS and healthy neutrophils.** Peripheral blood was drawn from patients (Multiple Sclerosis, MS; RRMS, red; SPMS, blue; or PPMS, grey) and healthy donors (HD, black). Neutrophils were isolated immediately via negative magnetic selection, centrifuged and cell pellets were snap-frozen. (A-G) Cell pellets were analysed via LC/MS for protein abundance. N values 6-8. Statistical test: unpaired t test with Welch’s correction.

Finally, we examined apoptosis-related proteins to determine whether neutrophils from patients with MS had increased life spans (Fig. 6A). We examined caspase 1 (CASP1, Fig. 6B), caspase 8 (CASP8, Fig. 6C), TRAIL (TNFSF10, Fig. 6D), BH3 interacting domain death agonist (BID, Fig. 6E), BCL2 associated X (BAX, Fig 6F) and apoptotic peptidase activating factor 1 (Fig. 6G); only one, BID, was found to be differentially expressed between our two groups of neutrophils, being less abundant in MS neutrophils.

### MS neutrophils have altered carbohydrate and lipid metabolism

We were interested in how proteins relating to metabolism and metabolic signalling pathways were altered in MS neutrophils since others have demonstrated altered neutrophil metabolism in chronic inflammatory conditions such as rheumatoid arthritis [10]. Neutrophil metabolism has generally been considered to be predominantly glycolytic, as these cells have relatively low mitochondrial content; however, more recently it has been demonstrated that certain neutrophil functions, including chemotaxis, NETosis and degranulation, require mitochondrial substrate oxidation (reviewed in [35–37]).

**Figure 7:**
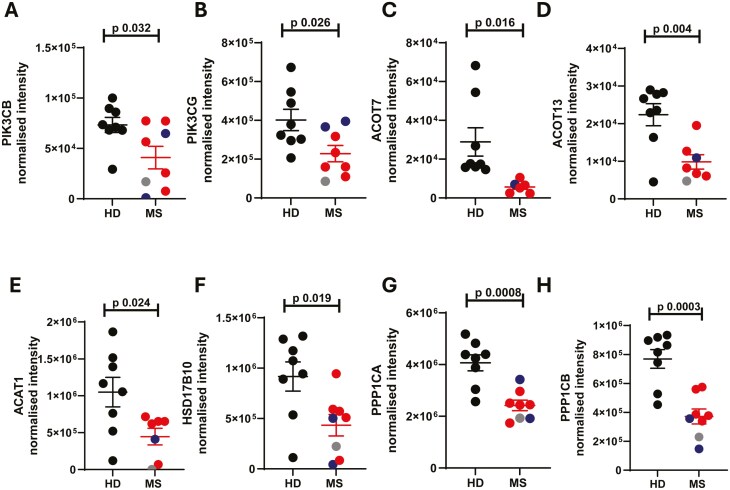
**Reduced activity of metabolic pathways in MS neutrophils compared to healthy control cells.** Peripheral blood was drawn from patients (Multiple Sclerosis, MS; RRMS, red; SPMS, blue; or PPMS, grey) and healthy donors (HD, black). Neutrophils were isolated immediately via negative magnetic selection, centrifuged and cell pellets were snap-frozen. (A-F) Cell pellets were analysed via LC/MS for protein abundance. N values: 6-8. Statistical test: unpaired t test with Welch’s correction.

The class I phosphoinositide 3-kinase (PI3K catalytic) subunits p110β/PIK3CB and p110γ/PIK3CG (Fig. 7A, B) were significantly less abundant in MS neutrophils compared to healthy controls. This major signalling pathway supports processes including phagocytosis [38] and NET formation [39]. Interestingly, neutrophils from mice lacking PI3K subunits and associated proteins have reduced ROS and dysregulated degranulation [40, 41], therefore reduced PI3K subunits in MS neutrophils could be linked to the reduction in ROS and altered degranulation observed.

In addition, members of the acyl-CoA thioesterase (ACOT) family pathway, which regulates fatty acid metabolism, intracellular trafficking and storage of fatty acyl-CoAs [42], were strongly decreased in abundance (Fig. 7C, D). Acetyl-CoA acetyltransferase 1 (ACAT1) and hydroxysteroid 17-beta dehydrogenase 10 (HSD17B10), involved in mitochondrial fatty acid beta-oxidation, were also significantly downregulated (Fig. 7E, F).

Finally, two catalytic subunits of protein phosphatase 1 (PP1) (PPP1CA and PPP1CB), which regulates glycogen breakdown by dephosphorylation of rate-limiting enzymes in this pathway [43] were also strongly reduced in MS neutrophils compared to healthy donors (Fig. 7G, H). This may be relevant since glycogen stores were recently shown to critically support neutrophil survival and function [43]. Taken together, these data indicate that MS neutrophils may demonstrate reduced activity of metabolic pathways including glycolysis, mitochondrial fatty acid oxidation and glycogen breakdown. However, it will be important to define this at a functional level, particularly since certain of these proteins have complex roles in the dynamic regulation of these pathways. For example, both impaired and excessive activity of the PI3K pathway, in the context of loss- and gain-of-function subunit mutations, is associated with dysregulated metabolism and function of immune cells (reviewed in [44]). The data may also reflect alterations in neutrophil maturation status, as immature neutrophils demonstrate higher mitochondrial content and dependency on lipid metabolism than mature counterparts (reviewed in [45]).

### MS neutrophils are less able to suppress T-cell activation

Neutrophils and T cells interact in the periphery, in lymph nodes, and in tissues including in the central nervous system [12, 13, 46]. We and others have previously shown that the interaction of resting neutrophils with T cells suppresses T cell proliferation and cytokine production [8, 47–49], while contact with primed neutrophils, by contrast, promotes T cell activation [47]. In particular, previous papers have indicated CD11b [8], cathelicidin [11], and elastase [50] can induce activation and differentiation of responding T cells, and these are all over-expressed in MS neutrophils in our dataset; in contrast, arginase is suppressive of T cells and this is not altered in our dataset. We therefore hypothesized that the increased activation of T cells seen in patients with MS may, in part, result from impaired tonic regulation by resting neutrophils, owing to the altered expression of T cell stimulating versus suppressive proteins.

We tested this hypothesis using a co-culture system we have previously established [47]. Neutrophils and T cells were rapidly isolated from peripheral blood and cultured together for 24 hours at a 1:3 T cell: neutrophil ratio, in the presence of anti-CD3/CD28/CD2 stimulation. The T cell phenotype was then assessed by flow cytometry. We compared MS neutrophil-MS T cell and HD neutrophil-HD T cell co-cultures and assessed the activation of the T cells.

**Figure 8:**
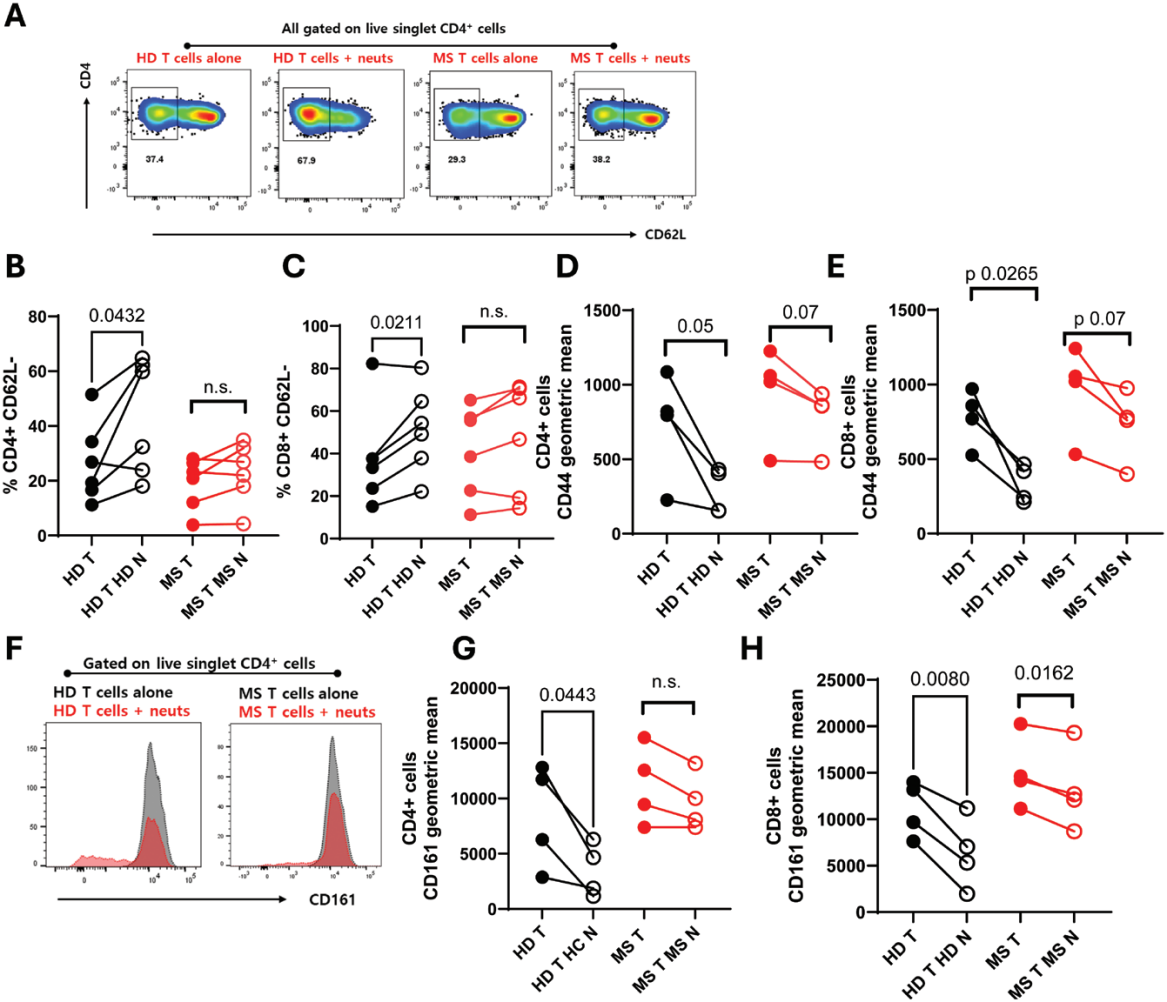
**MS neutrophils are less able to suppress T cells than healthy donor cells.** Peripheral blood was drawn from RRMS patients (MS, red) and healthy donors (HD, black). Neutrophils and total T cells were immediately isolated via negative magnetic selection. T cells were cultured either alone or with neutrophils (1:3 T:N) for 24 hours then assessed by flow cytometry. (A-E) activation of T cells was measured by CD62L and CD44 expression. (F-H) Expression of the Th17-related marker CD161 was quantified. N values: B, C – 6; D, E, G, H – 4. T = T cells alone, closed symbols; T N = T cells with neutrophils, open symbols. Statistical tests used – paired t test.

Here, co-culture with freshly isolated healthy neutrophils increased healthy T cell CD62L expression compared to T cells cultured alone (Fig. 8A–C), suggesting lower activation. This was true for CD4^+^ and CD8^+^ T cells in these cultures. However, MS neutrophils did not significantly alter the expression of CD62L on co-cultured T cells, indicating a reduced capacity to regulate T cell activity. Similarly, contact with healthy neutrophils also suppressed CD44 expression compared to T cells cultured alone (Fig. 8D,E), which, again, occurred to a much lower extent with MS neutrophils. Specifically, healthy neutrophils resulted in suppression of CD4^+^ T-cell CD44 expression to 38.9% of the original intensity, while MS neutrophils resulted in suppression only to 82.7% of the original intensity.

These data agree with previous studies which show that contact with resting neutrophils suppresses T cell activation, and extend these studies by demonstrating that this suppression is reduced with cells taken from MS patients.

CD161 expression on T cells is altered following neutrophil contact

Of particular interest to the T cell phenotype in MS is the expression of CD161. This surface molecule has shown to be a marker of IL-17-producing cells [51], and CD161^high^CD8^+^ cells are increased in the peripheral blood of MS patients [52], therefore we wondered whether neutrophils could regulate the expression of CD161 on T cells. After 24 hours of incubation, CD4^+^ T cells incubated with HD neutrophils showed a significant suppression in CD161 expression on their surface compared to those activated but with no neutrophil contact (Fig. 8G). In contrast, those incubated with MS neutrophils were not significantly suppressed.

CD8^+^ T cells from both donors were suppressed by autologous neutrophils (Fig. 8H), although the MS neutrophils suppressed the CD161 expression less (to an average of 86% of their original expression) than the HD neutrophils did (to an average of 54% of their original expression). Therefore, it is possible that MS neutrophils are less able to suppress CD161 expression on T cells *in vivo*, leading to the presence of increased numbers of CD161^+^ cells.

## Discussion

The aim of this study was to perform the first proteomic analysis of neutrophils from patients with MS, who are not on immune-modulating medication, to identify possible protein pathways that are dysregulated in MS neutrophils compared to healthy neutrophils.

We have shown here that neutrophils present in the circulation of MS patients are more activated and mature than in healthy controls, supporting previous studies performed using flow cytometry. One explanation for this could be that the increased inflammation and infectious burden in MS patients has triggered priming and activation of peripheral blood neutrophils. However, all of the patients showed increased maturity of the neutrophil populations, with a very consistent magnitude of increase. This suggests it is very unlikely that viral infections are responsible for the increased maturity as it is unlikely all patients simultaneously were suffering from viral infections, of similar magnitude.

We have also shown that MS neutrophils have significantly increased granule protein content compared to HD neutrophils. Neutrophils degranulate in reverse order—that is, the secretory granules are released first, then tertiary, secondary and finally primary granules. One explanation of our data is therefore that peripheral blood neutrophils in patients with MS contain higher levels of granule proteins, and also a decreased propensity to degranulate, so that only the tertiary granules have been released and the primary and secondary granules remain in the cells. However, the increased expression of CD63 on the surface of the MS neutrophils argues against this and suggests there is also an increased release of primary granules by these cells. While we are unclear as to the mechanisms of release, this study clearly shows that multiple granule proteins are increased in MS neutrophils; as we know that granule proteins have a profound impact on the infection status of patients as well as their adaptive immune responses [53], this is an important avenue to investigate. It is intriguing that MS neutrophils have such high concentrations of these antimicrobial proteins while the patients have increased infection risk. We hypothesize that dysregulated release of these proteins may explain this, or that the other pathways shown to be altered in our study (such as the MAVS pathway) affect the anti-infective capacity of these neutrophils in other ways. Previous studies in other conditions such as systemic lupus erythematosus have shown increased granule proteins in the serum of patients compared to healthy controls [54, 55]. However, this has been interpreted as increased degranulation by neutrophils, rather than the increased concentrations of these mediators to start with, which we see in our MS samples. We hypothesize that MS neutrophils have a disordered degranulation pattern compared to healthy controls as well as disordered production of granule proteins. A systematic analysis of combined neutrophil proteomics and secretomics in MS is now needed in a larger cohort of patients, to test this hypothesis and to understand the balance between degranulation and retention of granules. Future studies with a larger cohort will also specifically collect more patients with SPMS and PPMS as well as RRMS. We are intrigued by the SPMS patient here with extremely high granule peptide contents; with only two SPMS patients in this dataset, we cannot draw conclusions about this, but will properly power the collection of this data in new studies.

A limitation of this study is that we isolated all neutrophils rapidly using negative magnetic selection rather than by density centrifugation. As a result, we could not separate cells according to their density, and low- and normal-density neutrophil populations will be combined in the proteomic analysis. Patients with MS have been observed to have a small but consistent population of low-density neutrophils [56]. The low-density population is small enough that it would be unlikely to explain large alterations in protein content across neutrophils as a whole, as we see here. However, it would be very interesting to repeat the granule protein analysis in particular in normal-density and low-density neutrophils, to understand whether there is one neutrophil population which is particularly dysregulated in disease.

We demonstrated decreased expression of some ROS proteins in MS neutrophils. Naegele and Hertwig have separately shown an increased functional respiratory burst in neutrophils from patients with MS [17, 22]. Of note, in both of these papers, neutrophils were stimulated with fMLP and the mean fluorescence intensity (MFl) of oxidized dihydrorhodamine was measured. Therefore, it is possible that an increase in ROS is observed upon neutrophil stimulation only and not in neutrophils in a resting state (as the cells in this study were).

Our co-culture data demonstrates overall that healthy neutrophils suppress T-cell activation, but this does not occur with cells from MS patients. It is possible that the altered composition of MS neutrophils results in different outcomes for T cells, or that the dysregulation of MS T cells means they cannot respond to neutrophils properly. The answer may, of course, be both of these. Neutrophils can express HLA-DR and present antigens to T cells [57–61], so cross-donor cultures are unsuitable for unravelling the mechanisms behind the observations we make here. However, our data demonstrate that the neutrophil-T cell interaction is altered in MS compared to healthy donor cell cultures—a hugely interesting observation, as we are now aware of how important the interaction of neutrophils with T cells is for longer-term adaptive immune responses.

We also show that healthy neutrophils suppressed the expression of CD161 on T cells in co-culture, but MS neutrophils were unable to do so. This supports previous data showing that patients with MS have higher numbers of these CD161^+^ cells [52]. These cells are more likely to be IL-17-producing, suggesting that normal resting neutrophils have a role in suppressing differentiation of the Th17 subset of cells. In an added layer of complexity, this impact may be switched to a pro-inflammatory Th17-driving role when the neutrophils are primed, de-granulating or NETosing, as granule contents can induce Th17 differentiation [11, 50]. We note also that T cell release of IL-17—which occurs at higher levels in MS and is a key feature of the disease– is an inducer of granulopoiesis and of neutrophil maturation and activation [62, 63]. This may therefore be a positive feedback loop of increasing neutrophil maturation and IL-17 expression.

This study emphasizes the role of neutrophils and their interactions with T cells in MS pathology, highlighting the importance of unravelling the complex interplay of these two cell types in longer-term autoimmune disease.

## Data Availability

The proteomic dataset collected is available at FigShare, https://doi.org/10.6084/m9.figshare.24948342.v2
